# The causes of stillbirths in south Asia: results from a prospective study in India and Pakistan (PURPOSe)

**DOI:** 10.1016/S2214-109X(22)00180-2

**Published:** 2022-06-14

**Authors:** Elizabeth M McClure, Sarah Saleem, Shivaprasad S Goudar, Shiyam Sunder Tikmani, Sangappa M Dhaded, Kay Hwang, Gowdar Guruprasad, Dhananjaya Shobha, B Sarvamangala, S Yogeshkumar, Manjunath S Somannavar, Sana Roujani, Sayyeda Reza, Jamal Raza, Haleema Yasmin, Anna Aceituno, Lindsay Parlberg, Jean Kim, Carla M Bann, Robert M Silver, Robert L Goldenberg, PURPOSe Study Group, Shivaprasad Goudar, Sangappa M Dhaded, Mahantesh B Nagmoti, Manjunath S Somannavar, S Yogeshkumar, Gowdar Guruprasad, Gayathri H Aradhya, Naveen Nadig, Varun Kusgur, Chaitali R Raghoji, B Sarvamangala, Veena Prakash,, Upendra Kumar Joish, G K Mangala, K S Rajashekhar, Sunil Kumar, Vardendra Kulkarni, Sarah Saleem, Shiyam Sunder Tikmani, Afia Zafar, Imran Ahmed, Zeeshan Uddin, Najia Ghanchi, Shabina Ariff, Lumaan Sheikh, Waseem Mirza, Haleema Yasmin, Jamal Raza, Jai Prakash, Furqan Haider, Anna Aceituno, Lindsay Parlberg, Janet L Moore, Kay Hwang, Suchita Parepelli, Jean Kim, Carla Bann, Elizabeth McClure, Robert Goldenberg

**Affiliations:** a Research Triangle Institute International, Durham, NC, USA; b Department of Community Health Sciences, Aga Khan University, Karachi, Pakistan; c KLE Academy of Higher Education and Research, J N Medical College, Belagavi, Karnataka, India; d Department of Neonatology, Bapuji Educational Association's JJM Medical College, Davangere, India; e Department of Obstetrics, Bapuji Educational Association's JJM Medical College, Davangere, India; f National Institute of Child Health, Karachi, Pakistan; g Department of Obstetrics and Gynecology, Jinnah Postgraduate Medical Centre, Karachi, Pakistan; h Department of Obstetrics and Gynecology, University of Utah School of Medicine, Salt Lake City, UT, USA; i Department of Obstetrics and Gynecology, Columbia University, New York, NY, USA

## Abstract

**Background:**

South Asia contributes more than a third of all global stillbirths, yet the causes remain largely unstudied in this region. New investigations, including novel assessments of placental and fetal tissues, facilitate more precise determination of the underlying causes of stillbirth. We sought to assess underlying and contributing causes of stillbirth from settings in India and Pakistan.

**Methods:**

In this prospective cohort study (PURPOSe), we report the cause of death in stillbirths in hospitals in central India and south Pakistan (Davangere, India [three public and private hospitals] and Karachi, Pakistan [one public maternity and one children's hospital]). Women aged 15 years or older and with a known stillbirth (defined as a pregnancy at 20 or more weeks of gestation with the in-utero death of a fetus) weighing 1000 g or more were included in the study. Maternal clinical factors, placental evaluation, fetal tissue evaluation (from minimally invasive tissue sampling), and PCR for microbial pathogens were used to identify the causes of death. An expert panel reviewed available data for all stillbirths to identify the primary and contributing maternal, placental, and fetal causes of stillbirth.

**Findings:**

Between Sept 1, 2018, and Feb 12, 2020, 981 stillborns were included and, of those, 611 were reviewed by the expert panel. The primary maternal causes of stillbirth were hypertensive disease in 221 (36%) of 611 stillbirths, followed by severe anaemia in 66 (11%) stillbirths. The primary placental causes were maternal and fetal vascular malperfusion, in 289 (47%) stillbirths. The primary fetal cause of stillbirth was intrauterine hypoxia, in 437 (72%) stillbirths. We assessed the overlap of main causes and 116 (19%) stillbirths had intrauterine hypoxia, placental malperfusion, and eclampsia or pre-eclampsia indicated as primary causes of death. Infection (including of the placenta, its membranes, and in the fetus) and congenital anomalies also were causative of stillbirth.

**Interpretation:**

In south Asia, fetal asphyxia is the major cause of stillbirth. Several placental lesions, especially those associated with maternal and fetal vascular malperfusion and placental abruption, have an important role in asphyxia and fetal death. Maternal hypertension, and especially pre-eclampsia, is often the primary maternal condition associated with this pathway.

**Funding:**

Bill & Melinda Gates Foundation.

## Introduction

The south Asia region contributes to more than a third of the global burden of stillbirths—a disproportionately large number. Although advances have been made in reducing child mortality in this region, the numbers and rates of stillbirths remain high.^1^, ^2^, ^3^ The causes of stillbirth in this region, as in most low-income and middle-income countries (LMICs), are mostly identified with verbal autopsy or clinical determination.^4^, ^5^, ^6^ Compared with other causes of death, an accurate cause cannot often be identified through verbal autopsy or clinical observation, and, in some settings, more than 75% of all stillbirths have unknown causes.^4^, ^5^ These gaps in understanding the causes of stillbirth have probably contributed to the slow progress in reducing stillbirth incidence, especially in LMICs.

One tool to improve specificity when identifying the causes of stillbirth is minimally invasive tissue sampling (MITS; a process of fetal tissue sampling by needle biopsy^6^), which has been used as a less invasive autopsy for histological and microbiological evaluations on selected tissues.^7^ For stillbirths, histological evaluation of the placenta is also essential to establish an accurate cause of death, as various placental conditions have been identified as common and important contributors to stillbirth.^8^, ^9^ However, in low-resource settings, placental evaluation has rarely been used in stillbirth evaluations.^10^, ^11^, ^12^

From a previous comprehensive literature review, we concluded that most stillbirths in LMICs probably occur secondary to fetal asphyxia and are generally associated with pre-eclampsia or eclampsia, antepartum haemorrhage, fetal growth restriction, placental abruption and placental vascular malperfusion, prolonged or obstructed labour, uterine rupture, and umbilical cord accidents.^13^ One study that used MITS is the CHAMPS study,^12^ which was done in LMICs. In preliminary analyses of 180 stillborn babies, primarily in African countries, the most common underlying causes of stillbirth were identified to be perinatal asphyxia or hypoxia (72%) and congenital infection or sepsis (15%), although the placenta was not available for evaluation in most cases.^12^

**Research in context**

**Evidence before this study**

In low-income and middle-income countries (LMICs), where 98% of all stillbirths occur, stillbirths are under-reported and few reports are available on the cause. When reports on causes are available, the majority have been based on verbal autopsy, which is not precise in identifying the cause of stillbirths, and they report a large proportion of stillbirths with an unknown cause of death. In high-income countries, placental causes have been found to be a major contributing cause of stillbirths. Recent advanced investigations, such as minimally invasive tissue sampling and placental examinations, contribute to a more accurate understanding of the causes of death, especially in LMICs. Only a few studies from LMICs have used minimally invasive tissue sampling or other more accurate tools for cause-of-death assessment. One study on cause of death among 180 stillbirths, primarily from Africa, concluded that the majority were caused by intrapartum hypoxia, although little placental evaluation was done. A second pilot study from South Africa also found hypoxia as a major cause of stillbirth along with fetal infection.

**Added value of this study**

PURPOSe, one of the largest prospective studies of stillbirth from south Asia, included a detailed assessment of stillbirths, such as placental assessment, physical examination, and histological evaluation of fetal tissues. Placental and fetal tissues were also tested for a wide range of pathogens by PCR. Expert panellists reviewed all available data presented in standardised format to identify the primary and contributing maternal, placental, and fetal causes based on the WHO International Classification of Disease for perinatal mortality, 10th revision (ICD-10 PM) classification.

**Implications of all the available evidence**

The techniques used for this study can inform future efforts to more completely and accurately identify the cause of stillbirth in LMIC settings. This is an important advancement towards reducing the large burden of stillbirth in south Asia.

In 2018, PURPOSe was launched to improve estimates of the specific causes of stillbirth in India and Pakistan.^14^ PURPOSe used prospectively collected data on maternal clinical factors, placental evaluation using the Amsterdam Consensus Criteria,^12^ MITS, and PCR for microbial pathogens to inform cause of death.^13^ We analysed the primary causes of death using a standardised approach,^14^ and present the results here.

## Methods

### Study design and participants

PURPOSe was a prospective, observational study using standardised data collection methods in two sites in south Asia (Davangere, India [three public and private hospitals] and Karachi, Pakistan [one public maternity and one children's hospital]). The study sites were selected to represent two south Asian areas on the basis of the high rates of stillbirth in the geographical region and the experienced research teams.^14^, ^15^ Although the full study investigated the cause of death for both deaths of preterm neonates and stillbirths, in this Article, we report the results of the cause-of-death study for stillbirths.

At participating study hospitals, research staff screened all pregnant women at the time of presentation to hospital for delivery between Sept 1, 2018, and Feb 12, 2020, using a study screening log. Women with a known stillbirth, defined as a pregnancy at 20 or more weeks of gestation with the in-utero death of a fetus, were approached for enrolment in the study. Additionally, if a woman with a stillbirth was identified at or after delivery, she was recruited as soon as possible after delivery. Women aged 14 years or younger and those who did not provide consent were excluded.

The study was reviewed and approved by the ethics review committees at the Aga Khan University (Karachi, Pakistan), KLE Academy of Higher Education and Research (Belagavi, India), JJM Medical College (Davangere, India), and RTI International (Durham, NC, USA). All women were provided counselling about the study (including risks and benefits) before providing informed written consent for participation in the study. Additional consent was obtained for the specimen collection and evaluation. For women younger than the legal age per country policy, parental consent was obtained in addition to the woman's assent before enrolment. Information on the cause of death was not provided routinely but each participant was notified that the individual results would be shared with the family on request.

### Data collection

A trained data collection team recorded findings on study data forms at the time of delivery, including data from a physical examination, and procedures for the current delivery. Medical and obstetric history were recorded from medical data abstraction (when available) and maternal recall. At hospital admission, the presence of fetal heart tones was assessed and documented. Gestational age was identified using ultrasound, or, when ultrasound was unavailable, using last menstrual period and clinical assessment by the clinician with an algorithm based on American College of Obstetrics guidelines.^16^ Using standardised operating procedures, the placenta and cord blood were collected for all deliveries. Before delivery, a rectovaginal swab was also obtained from each pregnant woman for assessment of group B streptococcus infection. The placenta was evaluated by trained pathologists who used standard operating procedures based on the Amsterdam Consensus criteria.^17^

Each stillborn baby had a physical examination after delivery, including assessment of birthweight, length, head circumference, and evaluation of signs and degree of maceration. With additional consent from the woman, MITS, which included needle biopsies from the lung, liver, and brain tissues, was done by trained technicians using standardised procedures.^18^ All samples were collected in duplicate to allow for a back-up specimen and were tracked with study labels. These study samples were subsequently analysed by histological and molecular microbiological methods, using TaqMan Array Card (TAC) PCR testing by the research laboratory team.^19^ The TAC PCR methods were validated by the US Centers for Disease Control and included testing of more than 50 pathogens and toxins. PCR was done for the fetal tissues (collected by MITS) as well as blood and cerebral spinal fluid samples. The placenta was examined grossly and histologically, and PCR was done on placental, umbilical cord, and membrane tissues. For all specimen evaluations, central research staff trained on study procedures at each site and quality review was done throughout the study. At the India site, with additional parental consent, a standardised clinical perinatal autopsy was also done for a subset of stillbirths. The autopsy was done by a trained pathologist with oversight from a senior pathologist.

For cause-of-death identification, stillborns that had a birthweight of 1000 g or more, and for which the placental evaluation was completed, were evaluated by a panel of medical experts to identify the causes of death. We chose to focus on stillborns weighing 1000 g or more because these comprise the vast majority of stillbirths and were likely to have the most informative findings for responses. In addition, having complete clinical information, including the placental evaluation, was required to improve accuracy of findings, given the ability of the placental evaluation to help elucidate cause of death in more than 60% of stillbirth cases.^9^ In addition, the MITS examinations on stillborns weighing less than 1000 g were often not informative for identification of the cause of stillbirth.^20^

For each stillbirth, the primary maternal, placental, and fetal cause of death, as well as the contributing causes, were identified by a panel of experts using standard procedures.^15^, ^21^ The expert panel approach was used to provide a standardised approach to cause-of-death determination. The expert panels, which were established for each site, comprised obstetricians, paediatricians, pathologists, microbiologists, and other clinicians. For each stillbirth, a pair of panellists, who were not directly involved with the conduct of the study, reviewed the case report. Case reports included a brief clinical description of the case and all positive clinical maternal, fetal, and placental findings, and results of the PCR bacteriological investigation and MITS histology.^21^ Additionally, reference measures, including the tenth percentile for gestational age based on the INTERGROWTH-21st criteria^22^ and mean placental weight for gestational age were presented in a standardised data report. Each pair of panellists met to discuss their findings, with any discrepancies resolved through discussion with a third panel member.

The panellists determined a primary maternal cause of death, a primary placental cause of death, and then a primary fetal cause of death, if applicable, and the maternal and placental causes could inform the fetal cause of death. The primary and contributing causes of stillbirth were based on the International Classification of Disease for perinatal mortality, 10th revision (ICD-10 PM).^23^

### Role of the funding source

The study was funded by grants from the Bill & Melinda Gates Foundation. The funder provided initial inputs into the study design but had no role in data analysis or data interpretation.

## Results

**Figure 1:**
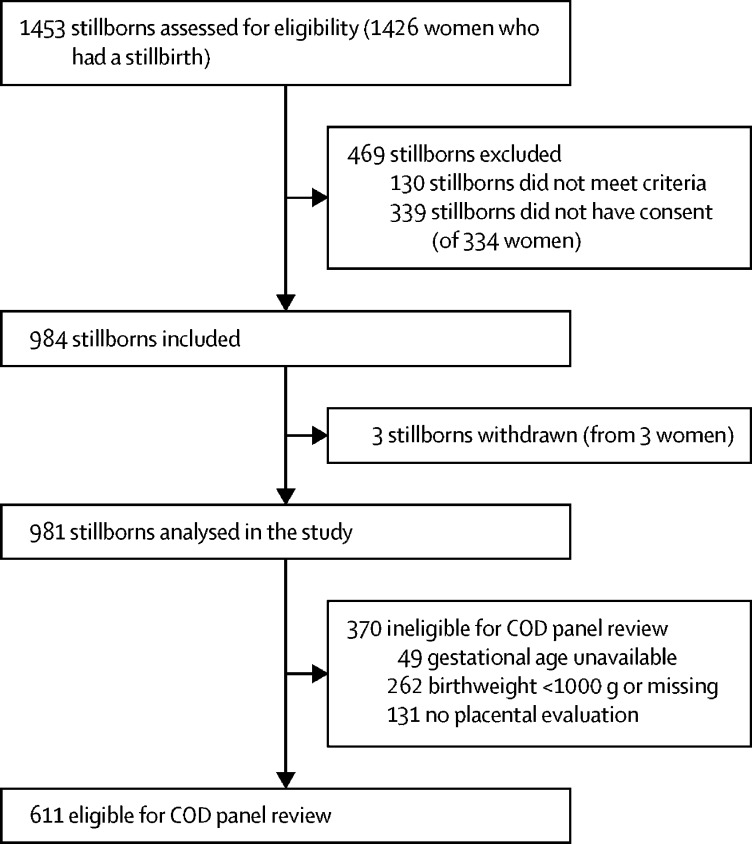
Selection of the study population COD=cause of death.

Between Sept 1, 2018, and Feb 12, 2020, a total of 1453 stillborns were screened and 1323 were eligible; of those, consent was provided for 984 (74·4%) stillborns and they were included in the study (figure 1). Of the 984 stillborns included, three were withdrawn from the study and 370 stillborns were excluded from the cause-of-death panel review (262 had a birthweight <1000 g, 131 did not have a placenta available, and 49 did not have the gestational age assessed). Thus, 611 (62%) of 984 stillborns delivered by 602 women met the eligibility criteria for the panel review and were included in the analyses of the cause of stillbirth.

Overall, 29 (5%) of 611 stillborns occurred at 20–27 weeks of gestation, 108 (18%) at 28–31 weeks of gestation, 260 (43%) at 32–36 weeks of gestation, and 214 (35%) at 37 or more weeks of gestation (table 1). 184 (30%) of 611 stillborns weighed less than 1500 g, 272 (45%) weighed 1500–2499 g, and 155 (25%) weighed 2500 g or more. At hospital admission, 482 (79%) of 611 stillborns were known to have died in utero (no fetal heart tones). On physical evaluation after delivery, 357 (58%) of 611 stillborns had signs of maceration. Distributions in birthweight and gestational age were similar between the Indian and Pakistani sites. More women were in their first pregnancy in the Indian site (113 [43%] of 263) than in the Pakistan site (93 [27%] of 348).

**Table 1:** Fetal, maternal, and placental characteristics

		**Overall (n=611)**	**India**	**Pakistan**
Stillborns with cause of death determination, n		611	263	348
Gestational age				
	20–27 weeks	29 (5%)	6 (2%)	23 (7%)
	28–31 weeks	108 (18%)	44 (17%)	64 (18%)
	32–36 weeks	260 (43%)	103 (39%)	157 (45%)
	≥37 weeks	214 (35%)	110 (42%)	104 (30%)
Birthweight				
	1000–1499 g	184 (30%)	82 (31%)	102 (29%)
	1500–2499 g	272 (45%)	113 (43%)	159 (46%)
	≥2500 g	155 (25%)	68 (26%)	87 (25%)
No fetal heart tone before delivery		482 (79%)	234 (89%)	248 (71%)
Macerated stillbirths		357 (58%)	162 (62%)	195 (56%)
Mothers of stillborns with COD determination, n		602	260	342
First pregnancy		206 (34%)	113 (43%)	93 (27%)
Multiple pregnancy		9 (1%)	2 (1%)	7 (2%)
Maternal age				
	<20 years	37 (6%)	19 (7%)	18 (5%)
	20–25 years	263 (44%)	156 (60%)	107 (31%)
	26–30 years	194 (32%)	64 (25%)	130 (38%)
	>30 years	108 (18%)	21 (8%)	87 (25%)
Maternal education				
	No formal schooling and illiterate	160 (27%)	44 (17%)	116 (34%)
	No formal schooling and literate	65 (11%)	8 (3%)	57 (17%)
	1–4 years	24 (4%)	13 (5%)	11 (3%)
	5–8 years	131 (22%)	71 (27%)	60 (18%)
	9–12 years	186 (31%)	102 (39%)	84 (25%)
	>12 years	32 (5%)	20 (8%)	12 (4%)
Any hypertensive disorder		238 (40%)	97 (37%)	141 (41%)
Antepartum haemorrhage		106 (18%)	49 (19%)	57 (17%)
Placenta evaluated, n		611	263	348
Mean placental weight, g (SD)		333·2 (131·5)	356·6 (128·1)	315·4 (131·4)
Presence of maternal malperfusion		355 (58%)	182 (69%)	173 (50%)
Presence of fetal malperfusion		118 (19%)	76 (29%)	42 (12%)
Inflammatory reaction		187 (31%)	79 (30%)	108 (31%)

Data are n (%) unless specified otherwise.

Overall, 602 women were included with a stillbirth for cause-of-death review and nine (1%) had a multiple birth. 263 (44%) of 602 women were aged 20–25 years (table 1). A substantially higher proportion of women were older than 30 years in Pakistan than in India (87 [25%] of 342 in Pakistan compared with 21 [8%] of 260 in India). The educational attainment of women also differed by site, with substantially more women in the Pakistani site being illiterate (116 [34%] of 342 compared with 44 [17%] of 260 in the Indian site). 238 (40%) of 602 women had a clinically identified hypertensive disorder and 106 (18%) of 602 had an antepartum haemorrhage. At the placental evaluation, maternal vascular malperfusion was identified in 355 (58%) of 611 cases, fetal vascular malperfusion in 118 (19%), and placental inflammation in 187 (31%).

Hypertensive diseases (primarily eclampsia and pre-eclampsia) were identified as the primary maternal cause of death for 221 (36%) of 611 stillbirths and a contributing cause for an additional 33 (5%; table 2). Severe maternal anaemia was identified as the main maternal cause in 66 (11%) of 611 stillbirths and a contributing cause for 199 (33%). Maternal infections were identified to be the primary maternal cause of stillbirth for 30 (5%) of 611 cases and contributed to an additional 52 (9%). Diabetes was considered the primary maternal cause for 22 (5%) stillbirths and contributed to an additional ten (2%) stillbirths. No primary maternal cause of death was identified for 200 (33%) of the 611 cases reviewed.

**Table 2:** Primary and contributing maternal causes of stillbirth identified by expert panellists

	**Primary maternal cause**			Contributing maternal causes*		
	Overall	India	Pakistan	Overall	India	Pakistan
Stillbirths with cause of death determination, N	611	263	348	611	263	348
Eclampsia, pre-eclampsia, or other hypertensive disorders	221 (36%)	94 (36%)	127 (36%)	33 (5%)	11 (4%)	22 (6%)
Maternal anaemia	66 (11%)	14 (5%)	52 (15%)	199 (33%)	75 (29%)	124 (36%)
Maternal infectious and parasitic disease	30 (5%)	13 (5%)	17 (5%)	52 (9%)	9 (3%)	43 (12%)
Diabetes	22 (4%)	7 (3%)	15 (4%)	10 (2%)	1 (0%)	9 (23%)
Preterm labour	2 (0%)	1 (0%)	1 (0%)	9 (1%)	6 (2%)	3 (1%)
Complications of caesarean section	1 (0%)	1 (0%)	0	0	0	0
Other maternal complications of pregnancy	38 (6%)	18 (7%)	20 (6%)	59 (10%)	11 (4%)	48 (14%)
Other complications of labour and delivery	31 (5%)	11 (4%)	20 (6%)	6 (1%)	3 (1%)	3 (1%)
No maternal cause identified	200 (33%)	104 (40%)	96 (28%)	310 (51%)	160 (61%)	150 (43%)

Data are n (%) unless specified otherwise.

* More than one contributing cause possible.

Placental, maternal, or fetal vascular malperfusion was the primary placental cause of death for 289 (47%) of 611 stillbirths and contributed to an additional 103 (17%) (table 3). Placental haemorrhage, including abruption or previa, was the primary cause of 94 (15%) of 611 stillbirths and contributed to an additional 21 (3%) stillbirths. Chorioamnionitis, funisitis, or another placental infection was identified to be the primary placental cause for 88 (14%) stillbirths and contributed to an additional 183 (30%) stillbirths. Only 83 (14%) of 611 stillbirths had no placental cause of death identified.

**Table 3:** Primary and contributing placental causes of stillbirth identified by expert panellists

	**Primary placental cause**			Contributing placental causes*		
	Overall	India	Pakistan	Overall	India	Pakistan
Placentas evaluated, N	611	263	348	611	263	348
Placental maternal or fetal vascular malperfusion	289 (47%)	121 (46%)	168 (48%)	103 (17%)	71 (27%)	32 (9%)
Placental abruption, haemorrhage, or previa	94 (15%)	45 (17%)	49 (14%)	21 (3%)	10 (4%)	11 (3%)
Chorioamnionitis, funisitis, or other infection	88 (14%)	43 (16%)	45 (13%)	183 (30%)	57 (22%)	126 (36%)
Cord complications	1 (0%)	0	1 (0%)	3 (0%)	0	3 (1%)
Other complications of placenta, cord, and membranes†	56 (9%)	15 (6%)	41 (12%)	100 (16%)	25 (10%)	75 (22%)
No placental cause identified	83 (14%)	39 (15%)	44 (13%)	259 (42%)	120 (46%)	139 (40%)

Data are n (%) unless specified otherwise.

* More than one contributing cause possible.

† Other complications included small placenta for gestation age (32 for primary placental causes and 32 for contributing placental causes) and haematoma (8 for primary placental causes and 28 for contributing placental causes).

The primary and contributing fetal causes of stillbirth as identified by the expert panel are summarised in table 4. Intrauterine hypoxia was the primary cause of death for 437 (72%) of 611 stillbirths and a contributing cause for an additional 63 (10%). Congenital infections were identified to cause 78 (13%) stillbirths and contributed to another 101 (17%). Congenital malformation was found to cause 23 (4%) stillbirths and contributed to an additional 14 (2%) stillbirths. Growth disorders, including small for gestational age, were identified by the panel as the primary cause of death of 21 (3%) stillbirths and contributed to another 237 (39%). For 46 (8%) of 611, no primary fetal cause of the stillbirth was identified.

**Table 4:** Primary and contributing fetal causes of stillbirth as identified by expert panellists

	**Primary fetal cause**			Contributing fetal causes*		
	Overall	India	Pakistan	Overall	India	Pakistan
Stillbirths with cause of death determination, N	611	263	348	611	263	348
Intrauterine hypoxia	437 (72%)	172 (65%)	265 (76%)	63 (10%)	40 (15%)	23 (7%)
Congenital infections	78 (13%)	41 (16%)	37 (11%)	101 (17%)	40 (15%)	61 (18%)
Congenital malformations, deformations, or chromosomal abnormalities	23 (4%)	11 (4%)	12 (3%)	14 (2%)	5 (2%)	9 (3%)
Growth disorders	21 (3%)	12 (5%)	9 (3%)	237 (39%)	108 (41%)	129 (37%)
Fetal haemorrhage	3 (0%)	1 (0%)	2 (1%)	0	0	0
Intraventricular haemorrhage of the fetus	1 (0%)	1 (0%)	0	3 (0%)	3 (1%)	0
Haemolytic disease of the fetus	1 (0%)	0	1 (0%)	1 (0%)	1 (0%)	0
Birth trauma	0	0	0	2 (0%)	0	2 (1%)
Other†	1 (0%)	0	1 (0%)	16 (3%)	6 (2%)	10 (3%)
No fetal cause identified	46 (8%)	25 (10%)	21 (6%)	254 (42%)	105 (40%)	149 (43%)

Data are n (%) unless specified otherwise.

* More than one contributing cause possible.

† Other conditions include seizure, hepatitis, fetal hydrops, high-grade glioma, rhesus haemolytic incompatibility, skin lesions, varicella zoster, meconium aspiration, polyhydramnios, twin–twin transfusion, and complications of prematurity.

The TAC PCR analyses were completed for 603 (99%) of the 611 placentas and for 441 (72%) of the 611 fetal tissues (appendix p 1). Of the more than 50 pathogens that were evaluated by TAC PCR, the most frequent pathogens identified in at least 2% of the placental tissue included *Ureaplasma urealyticum* (in 233 [39%] of 603 placentas), *Escherichia coli* (79 [13%] of 603), *Staphylococcus aureus* (47 [8%] of 603), *Candida albicans* (38 [6%] of 603), group B streptococcus (36 [6%] of 603), and *Enterococcus facium* (35 [6%] of 603). Similar pathogens were most frequently identified in the fetal tissues, but at lower rates, including *U urealyticum* (27 [6%] of 441 fetal tissues), *E coli* (21 [5%] of 441), *S aureus* (eight [2%] of 441), *Acinetobacter baumanii* (seven [2%] of 441), parvovirus B19 (seven [2%] of 441), and group B streptococcus (six [1%] of 441).

The presence of maceration was found in 357 [58%] of 611 stillbirths (appendix p 2). The most common primary maternal condition identified was hypertensive disorders in 118 (33%) of 357 stillbirths with maceration and 103 (40%) 254 stillbirths without maceration. Hypertensive disorders were followed by maternal anaemia (in 39 [11%] of 357 stillbirths with maceration and 27 [11%] of 254 stillbirths without maceration) and infection (in 22 [6%] of 357 stillbirths with maceration and eight [3%] of 254 stillbirths without maceration). Placental malperfusion was the most common placental condition for both groups and was identified in 185 (52%) of 357 stillbirths with maceration and 104 (41%) of 254 stillbirths without maceration. The second most common placental condition was placental infection, identified in 61 (17%) of 357 stillbirths with maceration and 27 (11%) of 254 stillbirths without maceration. Finally, for the primary fetal cause, intrauterine hypoxia was the most common cause in both groups but was higher in stillbirths without maceration (192 [76%] of 254) compared with stillbirths with maceration (245 [69%] of 357). The second most common fetal cause of death, congenital infection, was found in 57 (16%) of 357 stillbirths with maceration and 21 (8%) of 254 stillbirths without maceration.

**Figure 2:**
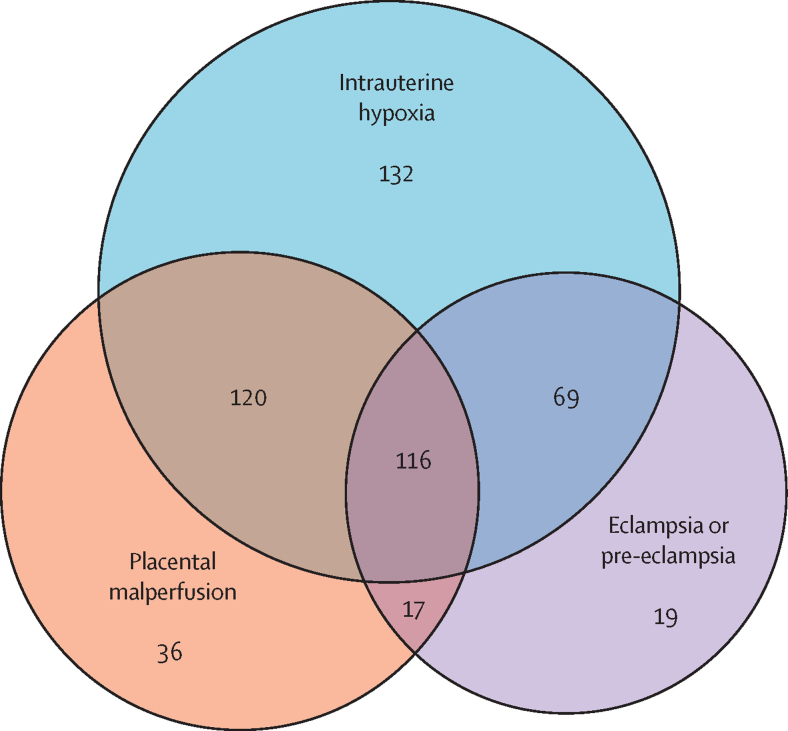
Overlap of the main placental, maternal, and fetal conditions

There was substantial overlap among the most common maternal, placental, and fetal conditions identified as primary causes of death (figure 2). The largest cluster, representing 116 (19%) of all 611 stillbirths, included eclampsia or pre-eclampsia, placental vascular malperfusion, and intrauterine hypoxia indicated as the primary causes of death. The second most common combination was eclampsia or pre-eclampsia, placental abruption, and intrauterine hypoxia, which occurred in 36 (6%) of 611 stillbirths. All other combinations represented less than 5% of the cases.

## Discussion

The PURPOSe study represents one of the largest prospective studies on the cause of stillbirth in south Asia. The most common primary fetal cause of stillbirth, as identified by the panel, was intrauterine hypoxia, followed by fetal infection and growth disorders. Hypertensive disease was the primary maternal cause (nearly a third of stillbirths), followed by anaemia and maternal infection. Placental and umbilical cord conditions were an important primary or contributing factor for more than 90% of all stillbirths, mostly involving placental malperfusion and haemorrhage. Various infections were also identified by the panel as either causing or contributing to an additional 30% of fetal deaths.

These findings point to common pathways for many of the stillbirths, such as maternal hypertensive disease together with placental fetal and maternal vascular malperfusion, and a small placenta, placental abruption, and fetal asphyxia accompanied by fetal growth restriction. Maternal and fetal infection contributed to about 10% of the stillbirths in these settings, whereas other pathways to stillbirth (eg, congenital anomalies and various maternal conditions) occurred less frequently.

This study represented one of the largest robust evaluations of the cause of stillbirths and included prospective data collection in two countries with a range of quality of care, and with high-quality historical, histological, and microbial evaluation to inform the assessments. Especially important was the inclusion of placental pathology, which was often crucial in determining cause of death. The panellists reviewed a standardised case report with results abstracted from clinical and research investigations. The availability of placental evaluations, which were assessed with standardised procedures based on the Amsterdam Consensus criteria,^12^ and the fact that the findings were similar across sites suggests the robustness of the findings. The main results for each site regarding conditions attributed to the cause stillbirth were similar, suggesting similar causality in both sites.

Because participants were recruited after delivery, the prenatal conditions were generally based on the mothers’ recall or review of medical records. For this reason, more detailed classification of hypertensive disorders was not possible. Another important limitation was the accuracy of gestational age. Since most women did not have early obstetric sonograms, the gestational age was often based on last menstrual period and obstetric evaluation, which has less precision than other methods.^16^ In addition, there might have been a meaningful interval between death of the fetus and delivery. Accordingly, the contribution of small for gestational age to the number of stillbirths could have been over-reported or under-reported. We restricted the study sample to fetuses weighing 1000 g or more, since babies born smaller than this are unlikely to survive in the settings where the study was done. Therefore, the causes among extremely preterm and small stillbirths, about 25% of the sample, were under-represented. Additionally, potential rare causes of stillbirth, such as those due to chromosomal anomalies and red blood cell disorders, were not routinely evaluated in this study. Finally, we acknowledge that non-medical and system-wide causes of stillbirth are important, but assessment of these issues was beyond the scope of this study.

Despite these limitations, this study adds to the understanding of the causes of stillbirth in south Asia. Most studies of the causes of stillbirth have reported a substantial proportion as due to an unknown cause.^10^, ^24^ We believe that, because many cases in this study had a MITS evaluation, TAC PCR evaluation for various organisms in multiple tissues, and a thorough placental evaluation, the proportion of stillbirths with an unknown cause was substantially reduced. A primary fetal cause of death could not be identified for only 8% of the stillbirths. Compared with the recent CHAMPS report on the cause of 180 stillbirths,^12^ with only 21 cases from the south Asia region, this study of 611 stillbirths with complete information substantially increases our knowledge about causes of stillbirth in the south Asian region.

The major findings of PURPOSe, which emphasise the confluence of maternal hypertensive conditions and placental malperfusion leading to growth restriction and asphyxia, have major implications for research and programmes that aim to reduce stillbirth.^25^ In almost all countries where stillbirth rates have been substantially reduced, the effective strategy has usually involved identifying fetuses at risk of stillbirth and delivering them before a fetal death.^26^, ^27^

Research is needed to develop interventions to interrupt the pathway that involves pre-eclampsia and placental damage, which leads to poor fetal growth and fetal asphyxia. Although maternal aspirin administration shows promise in reducing pre-eclampsia and fetal growth restriction, the extent to which it reduces stillbirth is unknown.^28^, ^29^ In the absence of a consistently effective prevention strategy, attention needs to be directed at developing better and more efficient tests to identify which fetuses are at the highest risk of death.

Currently, in high-income countries, most stillbirths are prevented with ultrasound to identify which fetuses are at risk because of growth restriction, a variety of tests to identify the fetuses most likely to die of asphyxia, and expedited delivery through labour induction or caesarean section, especially those occurring at or near term. Given increasing rates of institutional deliveries in south Asia,^30^ together with the rapid advancement of low-cost, evidence-based technologies, with appropriate, early identification of women with fetuses at risk, there is substantial opportunity to reduce stillbirths in the region.

## Data sharing

The data with deidentified participant data, supporting documents including the study protocol, case report forms, and informed consent forms are available on request from corresponding author with data access agreement.

## Declaration of interests

We declare no competing interests.
