# Supplementary material for: The causes of stillbirths in south Asia: results from a prospective study in India and Pakistan (PURPOSe)
Source: Lancet Glob Health. 2022 Jun 14;10(7):e970–7. doi: 10.1016/S2214-109X(22)00180-2 (PMC9210259; doi:10.1016/S2214-109X(22)00180-2)
Supplement: Supplementary appendix [file mmc1.pdf]

THE LANCET
Global Health

Supplementary appendix

This appendix formed part of the original submission and has been peer reviewed. We post it as supplied by the authors.

Supplement to: McClure EM, Saleem S, Goudar SS, et al. The causes of stillbirths in south Asia: results from a prospective study in India and Pakistan (PURPOSe). *Lancet Glob Health* 2022; **10:** e970–77.

Most common pathogens in placental samples and cord blood		Most common pathogens in fetus*	
**PCR Results Available, n/N**	603/611 (98·7)	**PCR Results Available, n/N**	441/611 (72·2)
Ureaplasma urealyticum and/or parvum	233 (38·6)	Ureaplasma urealyticum and/or parvum	27 (6·1)
Escherichia coli and/or Shigella species	79 (13·1)	Escherichia coli and/or Shigella species	21 (4·8)
Staphylococcus aureus	47 (7·8)	Staphylococcus aureus	8 (1·8)
Candida albicans	38 (6·3)	Acinetobacter baumanii	7 (1·6)
Streptococcus agalactiae (Group B Streptococcus)	36 (6·0)	Parvovirus B19 (human parvovirus)	7 (1·6)
Enterococcus facium	35 (5·8)	Streptococcus agalactiae (Group B Streptococcus)	6 (1·4)
Klebsiella pneumoniae	27 (4·5)	Human cytomegalovirus	5 (1·1)
Human cytomegalovirus	20 (3·3)	One or more species within the Rickettsia genus	5 (1·1)
Acinetobacter baumanii	19 (3·2)	One or more enterovirus serotypes with the Enterovirus genus	4 (0·9)
Parvovirus B19 (human parvovirus)	10 (1·7)	Klebsiella pneumoniae	4 (0·9)

*Samples include liver, lung (right & left), brain/central nervous system, cerebral spinal fluid, and whole blood.

Supplemental Table S1. Pathogens identified by TAC PCR

	Macerated	Non-macerated
**Stillbirths with cause of death determination, n/N**	357/611	254/611
**Maternal primary cause**	357	254
Eclampsia/preeclampsia or other hypertensive disorder, n (%)	118 (33·1)	103 (40·6)
Maternal anemia, n (%)	39 (10·9)	27 (10·6)
Maternal infectious and parasitic disease, n (%)	22 (6·2)	8 (3·1)
Diabetes, n (%)	15 (4·2)	7 (2·8)
Preterm labor, n (%)	2 (0·6)	0 (0·0)
Complications of caesarean section, n (%)	1 (0·3)	0 (0·0)
Other maternal complications of pregnancy, n (%)	22 (6·2)	16 (6·3)
Other complications of labor and delivery, n (%)	6 (1·7)	25 (9·8)
No maternal cause identified, n (%)	132 (37·0)	68 (26·8)
**Placental primary cause**	357	254
Placenta malperfusion, n (%)	185 (51·8)	104 (40·9)
Chorioamnionitis and/or funisitis, n (%)	61 (17·1)	27 (10·6)
Placenta previa or abruption, n (%)	29 (8·1)	65 (25·6)
Cord complication, n (%)	0 (0·0)	1 (0·4)
Other complications of placenta, cord and membranes[2], n (%)	34 (9·5)	22 (8·7)
No placental cause identified, n (%)	48 (13·4)	35 (13·8)
**Fetal primary cause**	357	254
Intrauterine hypoxia, n (%)	245 (68·6)	192 (75·6)
Congenital infections, n (%)	57 (16·0)	21 (8·3)
Congenital malformations, deformations or chromosomal abnormalities, n (%)	8 (2·2)	15 (5·9)
Growth disorders, n (%)	10 (2·8)	11 (4·3)
Fetal hemorrhage, n (%)	2 (0·6)	1 (0·4)
Intraventricular hemorrhage of the fetus, n (%)	1 (0·3)	0 (0·0)
Hemolytic disease of the fetus, n (%)	0 (0·0)	1 (0·4)
Other, n (%)	0 (0·0)	1 (0·4)
No fetal cause identified, n (%)	34 (9·5)	12 (4·7)

*Samples include liver, lung (right & left), brain/central nervous system, cerebral spinal fluid, and whole blood.

Supplemental Table S2. Primary causes of death by maceration status

We thank the PURPOSE Cause of Death Panelists

Panelist	***Institution***
Dr. Saba Tanveer Khan	Jinnah Postgraduate Medical Centre, Karachi, Pakistan
Dr. Maharukh Hyder	Jinnah Postgraduate Medical Centre, Karachi, Pakistan
Dr. Sidrah Nausheen	Aga Khan University, Karachi Pakistan
Dr. Shazia Masheer	Aga Khan University, Karachi, Pakistan
Dr. Atika Sher	Liaquat National Hospital, Karachi, Pakistan
Dr. Syeda Nazish Azim	Liaquat National Hospital, Karachi, Pakistan
Dr. Shaheen Taq	Abbasi Shaheed Hospital, Karachi, Pakistan
Dr. Sumaira Wajid	National Institute of Child Health, Karachi, Pakistan
Dr. Mehmood Shaikh	National Institute of Child Health, Karachi, Pakistan
Dr. Mohammed Hanif	National Institute of Child Health, Karachi, Pakistan
Dr. Yeshita V Pujar	KLE Academy of Higher Education and Research (Deemed-to-be-University)'s J N Medical College, Belagavi, India
Dr. Pralhad Kushtagi	CAO Durga Sanjeevani Manipal Hospital, Karnataka, India
Dr. Gowri Doriarajan	Jawaharlal Nehru Institute of Postgraduate Medical Education and Research (JIPMER), Dhanvantari Nagar, India
Dr. Shoba S Kumar	Tamilnadu Health Systems Project (TNHSP), Tamilnadu, India
Dr. Geetanjali Katageri	S Nijalingappa Medical College, Bagalkot, India
Dr. G Anuradha	St. John's Medical College and Hospital, Bangalore, India
Dr. Akhila Vasudev	Kasturba Medical College, Manipal, Karnataka, India
Dr. Philomena Vaz	Manipal Hospital, Karnataka, India
